# Exquisitely-preserved, high-definition skin traces in diminutive theropod tracks from the Cretaceous of Korea

**DOI:** 10.1038/s41598-019-38633-4

**Published:** 2019-02-14

**Authors:** Kyung Soo Kim, Martin G. Lockley, Jong Deock Lim, Lida Xing

**Affiliations:** 1 Department of Science Education, Chinju National University of Education, 3 Jinnyangho-ro 369beon-gil, Jinju-si, Gyeongnam, 52673 Korea; 2 Dinosaur Trackers Research Group, University of Colorado Denver, P.O. Box 173364, Denver, CO 80217 USA; 3 Cultural Heritage Administration, Government Complex-Daejeon, 189, Cheongsa-ro, Seo-gu, Daejon, 35208 Korea; 4 School of the Earth Sciences and Resources, China University of Geosciences, Beijing, 100083 China

**Keywords:** Palaeontology, Solid Earth sciences

## Abstract

Small theropod tracks, ichnogenus *Minisauripus*, from the Jinju Formation (Cretaceous) of Korea reveal exquisitely preserved skin texture impressions. This is the first report for any dinosaur of skin traces that cover entire footprints, and every footprint in a trackway. Special sedimentological conditions allowed footprint registration without smearing of skin texture patterns which consist of densely-packed, reticulate arrays of small (<0.5 mm) polygons, preserved as both impressions and casts, the latter essentially foot replicas. The skin texture resembles that reported for two Lower Cretaceous avian theropods (birds) from China which had quite different foot morphologies. This is also the oldest report of *Minisauripus* from Korea predating five reports from the Haman Formation of inferred Albian age. *Minisauripus* is now known from six Korean and three Chinese localities, all from the Lower Cretaceous. This gives a total sample of ~95 tracks representing ~54 trackways. With >80% of tracks <3.0 cm long, *Minisauripus* is pivotal in debates over whether small tracks represent small species, as the database suggests, or juveniles of large species.

## Introduction

The diminutive theropod track ichnogenus *Minisauripus* is represented by two ichnospecies. It was first described from Sichuan Province China^1^, as ichnospecies *M. chuanzhuensis*, from the Upper Cretaceous Daergun Formation of the Chiating Group, referred to as Late Cretaceous in age^2^, but elsewhere considered part of the Lower Cretaceous Jiaguan Formation^3^. *Minisauripus* was subsequently discovered at two sites in the Early Cretaceous Haman Formation (Hayang Group) of Korea and another site in the Early Cretaceous of Tianjialou Formation of Shandong Province, China^4^.

Finds in the 2000s indicated that the *Minisauripus* track was not made by a miniature blunt toed ornithopod as had initially inferred^1^ but was instead made by a theropod which often registered sharp claw traces, and often clearly showed two phalangeal pads on digit II and three on digit III, diagnostic of theropods. Clearly defined phalangeal pad traces were not observed on digit IV. Due to these diagnostic morphological features the new ichnospecies *M. zhenshounani* was erected on the basis of the Shandong material^4^, which included tracks somewhat longer than those reported from other sites: i.e., with footprint length (FL) up to 6.1 cm, including distinctive fine claw traces, whereas >80% of the global sample was in the FL range of 1.1–2.9 cm.

Since 2008, *Minisauripus* isp. has been discovered at a number of other Korean localities, all in the Haman Formation^5^, and at a new site in the Feitianshan Formation of Sichuan Province China^6^. The former, 2012 paper summarized the total *Minisauripus* database as consisting of “more than 80 well-preserved tracks comprising a minimum of 50 trackways from two localities in China and five in Korea”^5^: i.e., a total of seven localities. One of the localities, Buyun-ri consisted of “Five separate localities, in very close proximity”^5^. The addition of the new Chinese site increased the database so that “a total of ~92 *Minisauripus* tracks, representing at least 55 trackways, are now recorded from a total of eight localities”^6^ exclusive of sites in very close proximity which fall geographically within single key site locations. Clearly this constitutes a large multi-site sample of diminutive tracks, which bear only slight resemblance to larger theropod tracks assigned to quite different ichnotaxa (Suppl. Info).

**Figure 1:**
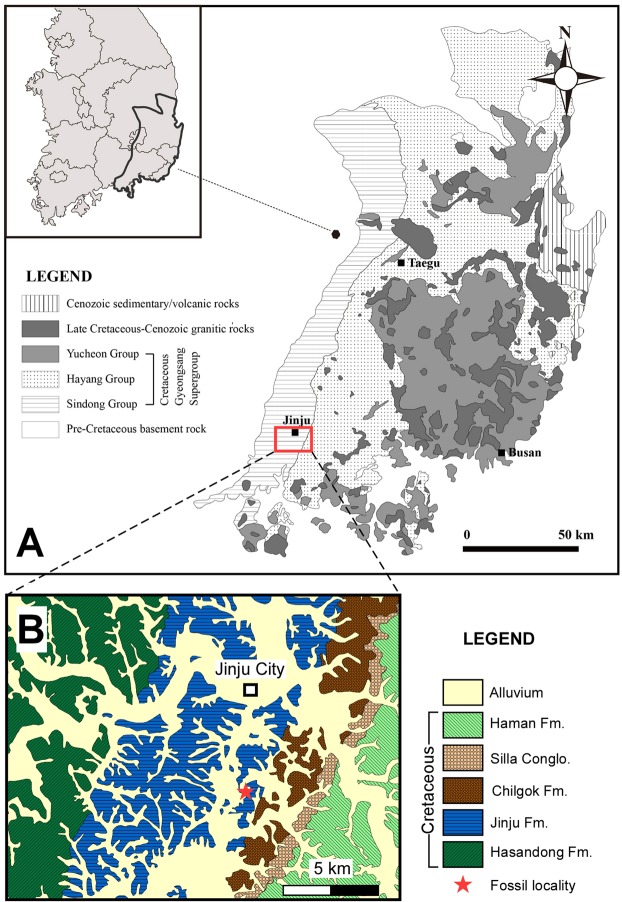
(**A**) Geological map of Gyeongsang Basin, in southeast sector of Korean peninsula (inset), showing outcrop of group-level units. (**B**) Geological map of formations around Jinju City showing fossil locality ~5 km south of the city center. Maps made by K-S K in Adobe photoshop (version CS6 www.adobe.com/Photoshop) and Canvas X (version, 2017 Build 160, http://www.canvasgfx.com/).

The present study, adds significantly to the growing data on *Minisauripus* morphology and distribution in space and time by adding well-preserved material, with exquisitely-preserved high-definition skin traces, from a ninth site, from the Early Cretaceous Jinju Formation in the Jinju City area of Korea (Fig. 1). The Jinju Formation in this area has recently attracted global ichnological attention for having produced large volumes of new track specimens 7 including a number of important new ichnotaxa, attributable to major groups such as mammals^7–11^, frogs^8^ and lizards^11^ which had not previously been reported from the Cretaceous of Korea. In addition diminutive tracks of purported microraptorine affinity have been described^9^.

This new *Minisauripus* occurrence described here is the first Korean site not associated with the younger Haman Formation (Fig. 1), and it is also the first from any global site to reveal *Minisauripus* skin impressions. Moreover, these skin traces are extraordinarily well preserved, and the only example known for such a small dinosaur. Thus, the material extends the known stratigraphic range of *Minisauripus* in Korea and gives us a high definition snapshot revealing the texture and ornamentation of the foot integument, which resembles a living foot replica, and can be compared with that reported from other theropod tracks.

## Material and Methods

The *Minisauripus* material described here, originates from major excavations in the Jinju Innovation City area (Fig. 1), at the Ppuri Industrial Complex, not far from the highly productive outcrops associated with the Korea National Natural Monument Number 534 project (named *The Pterosaur, Bird and Dinosaur Tracksite of Hotan-dong*), and the newly built *Jinju Pterosaur Footprint Museum* which already contains a number of recently described type and figured specimens^7–11^ from the Jinju Formation.

**Figure 2:**
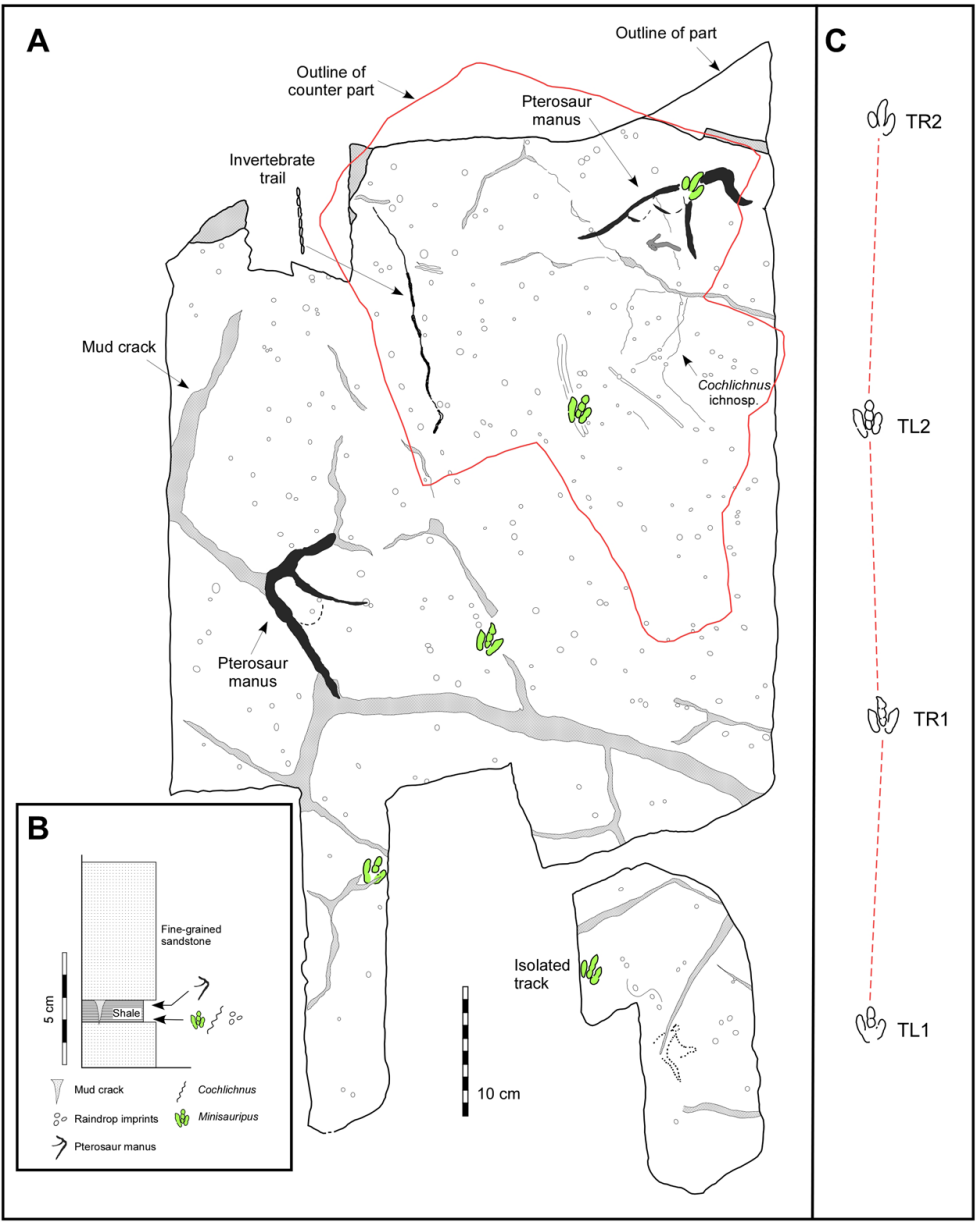
(**A**) Counterpart slab CUE JJ_M01 showing trackway with four consecutive *Minisauripus* track casts TL1-TR2. (**B**) Natural impression slab (CUE JJ_M02) showing tracks TL2 and TR2. (**C**) Isolated track specimen (CUE JJ_M03). Compare with Fig. 3. Photographs by K-S Kim and layout created in Canvas X (version, 2017 Build 160, http://www.canvasgfx.com/).

**Figure 3:**
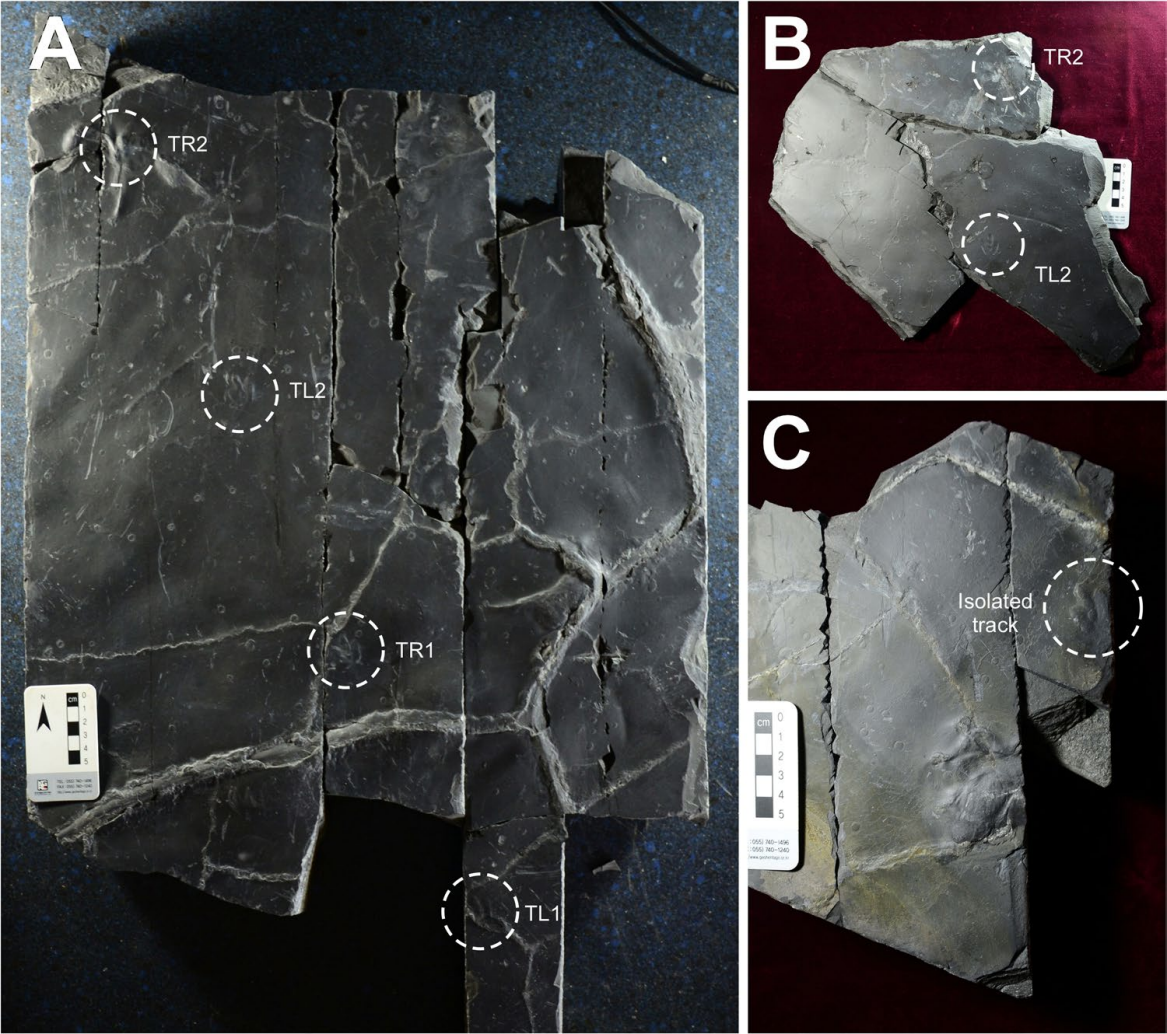
(**A**) Map of specimens CUE JJ_M01-3 showing four-track *Minisauripus* trackway, and additional isolated fifth track on small unconnected slab. Map based on counterpart cast of track-bearing surface. Red outline shows part of surface preserved as natural impressions. Pterosaur manus tracks, desiccation cracks (stippled areas), raindrop impressions and invertebrate traces also shown. Compare with Fig. 2 and text for details. (**B**) Shows microstratigraphy of part and counterpart of track-bearing slab. (**C**) Shows four track-trackway with dashed line to highlight steps and pace angulations. See Table 1 for measurements. Map made by M. G. Lockley and K-S Kim with layout created in Canvas X (version, 2017 Build 160, http://www.canvasgfx.com/).

The specimens are Chinju National University of Education (CUE) JJ_M01-M03 (Figs 2–3) and consist of a part and counterpart (M01 and M02) comprising a thin (~2.0 cm thick) slab of very fine grey sandstone with a dark grey to black mudstone drape less than 1.0 mm thick (Fig. 2B), with a four track-trackway (Figs 2A,B and 3B), as well as an isolated cast (M03), with a single track, from the same surface which, based on size and mode of preservation, likely belongs to the same trackway. The slab with the natural impressions (Fig. 2B) reveals two *Minisauripus* tracks from the same trackway and one pterosaur manus track in concave epirelief. There are also a few faint invertebrate traces, including very small *Cochlichnus* trails, and many small raindrop impressions between 2.0 and 5.0 mm in diameter. Replicas of casts TR1-TR2, impression TL2 and the isolated M03 track were made for comparative study as University of Colorado Museum of Natural History (UCM) replicas UCM 214.323, 324 and 325 respectively.

The 7.0 cm-thick counterpart slab (CUE JJ_M01) which originally overlay the clay-draped unit shows another ~1.0 cm of dark grey to black mudstone overlain by ~6.0 cm of fine-grained sand. The whole counterpart slab, with track casts, is larger (~50 × 50 cm) than the aforementioned slab with impressions, and reveals a sequence of four *Minisauripus* tracks comprising a single narrow trackway (Figs 2A,3B,C) in negative (convex hyporelief), and an additional pterosaur manus track. The density of raindrop impressions is estimated at 1000/m^2^. The raindrop impressions and the *Minisauripus* are very shallow and flat bottomed. These physical features of both the biogenic and non-biogenic traces give clues as to how the substrate conditions facilitated superior preservation.

Neither the part or counterpart slabs were found intact. When the first *Minisauripus* track was observed, in association with a surface containing raindrop impressions, excavation was halted while the research team collected all pieces with similar raindrop markings and tracks. It was then possible to reconstruct the part with two tracks and the corresponding counterpart with the four-track trackway. A fifth track was found, in negative hyporelief, but this has not been connected to the trackway. Thus, the sample consists of one four-track trackway cast, two impressions that match the two more distal tracks, and an isolated track cast. The trackway sequence was numbered TL1, TR1, TL2 and TR2 (with L and R indicating right and left). Individual track length, width, step, stride, pace angulation and trackway width were measured to the nearest millimeter (Table 1).

**Table 1:** Morphometric parameters, with means (in bold), for tracks in four-track *Minisauripus* trackway TL1-TR2, and for isolated *Minisauripus* track (t).

Track #	Length	Width	L/W	Step	Stride	T width	Pace angle
TL1	2.4	2.0	1.20	—	—	—	—
TR1	2.4	2.0	1.20	20.8	40.4	2.9	172°
TL2	2.4	1.9	1.26	19.6	38.8	2.7	174°
TR2	2.3	1.8	1.28	19.7	—	—	—
**means**	**2.38**	**1.93**	**1.23**	**20.03**	**39.6**	**2.8**	**173°**
Isolated t	2.3	1.3	1.28	—	—	—	—

T width = Trackway width.

**Figure 4:**
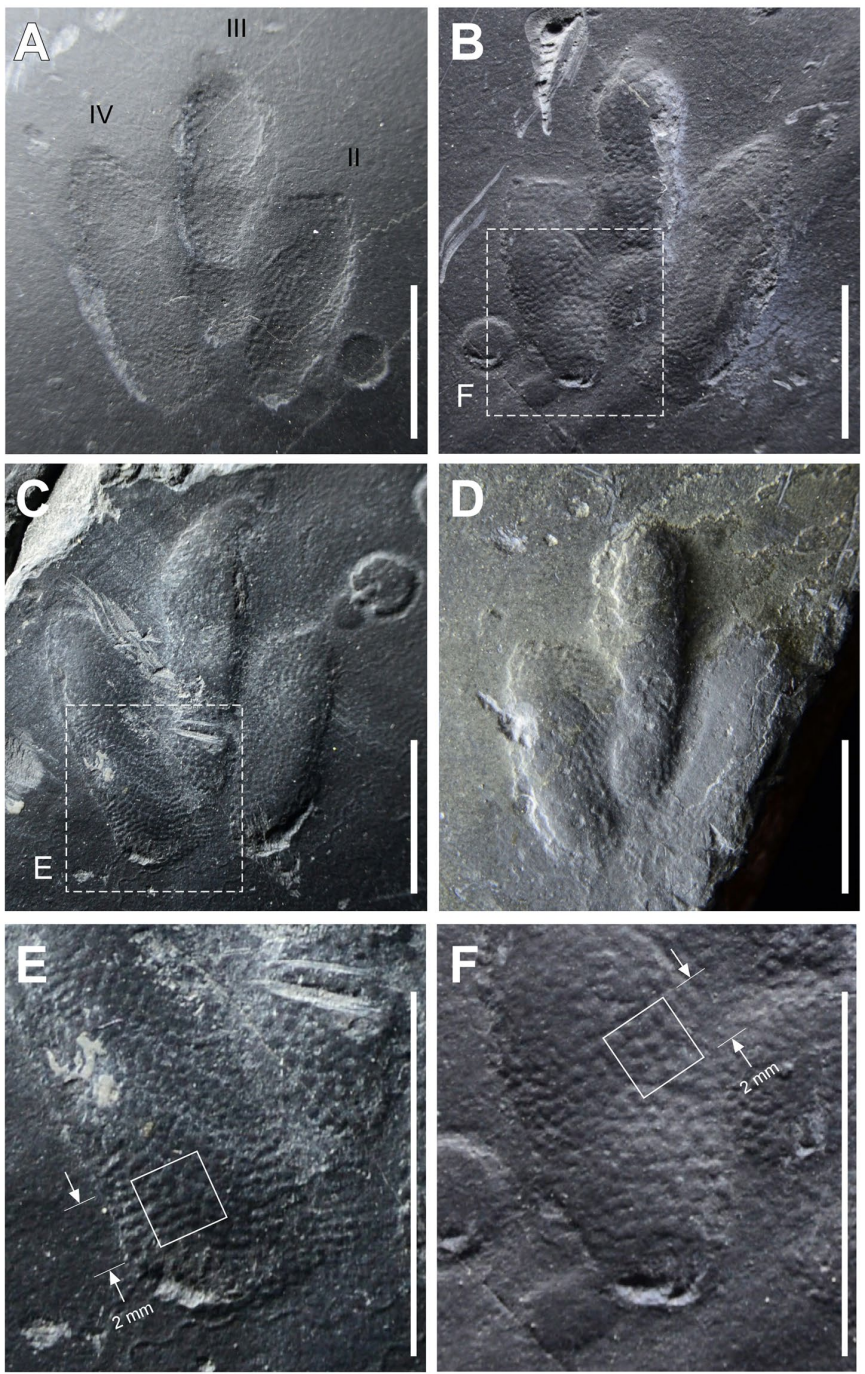
(**A**,**B**) Natural impression (**A**) and cast (**B**) of track T L 2 showing area enlarged in frame F. Note skin traces in hypex area between digits II and III. (**C**) Natural cast of track T R1, showing area enlarged in frame E. Note narrow, digit II intersecting raindrop impressions. (**D**) Isolated track t. Note skin traces in hypex area between digits II and III. E and F details of skin trace ornament in 2.0 × 2.0 mm areas of digits IV and II respectively from tracks TL2 and TR1. Casts show in frames B-F are essentially replicas of the living foot. Photographs by K-S Kim and layout created in Canvas X (version, 2017 Build 160, http://www.canvasgfx.com/).

All macroscopic details of the surfaces were traced on clear acetate film (Fig. 2A) and numerous photographs of the *Minisauripus* tracks were taken with low angle light (Fig. 4) in order to highlight the skin impression traces. Enlarged photographs of the individual *Minisauripus* tracks were examined minutely to analyze and measure the texture and patterning of the well-preserved skin impressions (Fig. 4).

## Description of Material

### Macromorphology of *Minisauripus*

As shown in Figs 2–3, and Table 1 the four-step *Minisauripus* trackway is narrow and very well preserved except where track TR2 was overprinted by a pterosaur track. The mean length of the four *Minisauripus* tracks is 2.38 cm and 1.93 cm (L/W 1.23) and the mean step and stride lengths are 20.03 cm and 39.60 cm respectively. Thus the step lengths were quite regular and the mean trackway width (2.8 cm) and the mean pace angulation (173°) reflect the narrowness and regularity in step length.

### Skin traces

In all five of the *Minisauripus* skin traces cover the entire surface where the footprint registered. This applies to both part and counterpart in the cases where both were recovered. Such consistent ‘coverage’ is unprecedented for any known dinosaur track occurrences. In the case of tracks TL1-TL2, because they are natural casts, they essentially present a ‘perfect’ replica of the living foot. As noted below this has significant implications for understanding the preservation of skin traces. The skin traces consist of very fine regular tubercles or protuberances in polygonal arrays, which give the sediment a patterned, sandpaper-like, woven or reticulate texture. The individual tubercles, convex mounds in the casts and concave dimples in the impressions, are about up to 0.5 mm in diameter. Under different illumination conditions the tubercles appear to be in distinct linear arrays reflecting the overall regularity of the original skin texture.

An interesting feature of most of the tracks is that they clearly show skin traces between the traces of digits II and III (Fig. 4) in the case of track TL2 (Fig. A,B) and isolated track t, (Fig. 4D), most of the area between the distal tip of digit II and the creases between the distal and the medial phalangeal pad of digit III is occupied by skin traces which show the polygonal array pattern registered continuously in the hypex space between digits II and III. The skin trace pattern in the hypex space between digits III and IV is not so clear in these two tracks, but does appear continuous in the hypex between digits III and IV of track TR1. There appear to be two explanations for these inter digital skin traces: 1) the skin was in fact a morphological feature connecting the digits in the more distal regions of the hypicies, or 2) the skin, and flesh between skin and bone was loose and pliable and easily pushed down as the foot registered so as to impress in the hypex space. Arguably, in either case the looseness and flexibility of the skin may have been a factor in allowing it to spread when contacting with the substrate so as not to shift or slide and smear the fine skin traces as they were registered.

### Pterosaur tracks

Two pterosaur manus tracks are preserved on CUE JJ_M01. Both are quite large, 14.0 cm long and ~7.0 cm wide in the case of the track that overlaps the *Minisauripus* track TR2, and 13.0 cm long and 8.0 cm wide in the case of the track to the left of TR1 (Fig. 3A), which is aligned with desiccation cracks suggesting that the cracks were biogenically induced. No associated pes tracks were observed and the tracks are not oriented in the same direction, so probably not part of a single trackway. However, both tracks show convex outward bulges between the traces of digits II and III which may be web traces. Although the pterosaur tracks are not of central interest to this report, as noted below, their preservation is of interest in comparison with the *Minisauripus* tracks.

## Discussion

The most significant results of the present study pertain to: (1) the evidence that *Minisauripus* is not confined to the Haman Formation in Korea and thus has a longer stratigraphic range than previously inferred; (2) the sample gives us the first detailed insight into the skin texture of a diminutive theropod, essentially replicating the soft tissue ornamentation; (3) the role of skin in revealing locomotor dynamics during registration of footprints, and; (4) the significance of the Jinju and other *Minisauripus* samples, in paleobiological debates over the implications of footprint evidence for diminutive size in theropod trackmakers. Each topic is briefly reviewed below.

The Jinju Formation has recently proved a rich source of new ichnotaxa, some new to the global record, such as the mammal track *Koreasaltipes jinjuensis*^7^, the diminutive dromaeosaurid tracks *Dromaeosauriformipes rarus*^9^, and some representing first occurrences in Korea, e.g., *Corpulentapus* isp. not previously known outside China^10^. Other newly discovered ichnites include a new lizard morphotype resembling, but different from, *Neosauroides koreaensis* from the Haman Formation^11,12^. These all come from within or near the large excavations undertaken in the immediate vicinity of the aforementioned Natural Monument Number 534, within Jinju City, and add significantly to other reports from the Jinju Formation in the wider region (~30 km radius of Jinju City). It is currently inferred that the Jinju Formation represents Aptian deposition between ~120 and ~112 mya, whereas the Haman Formation represents the Albian stage between ~112 and ~100 mya^13^. Since all previous Korean reports of *Minisauripus* are from the Haman Formation we can infer, if these age estimates are correct, that the Jinju occurrence likely represents track making activity some 10–20 million years prior to the activity of the Haman Formation trackmakers. The Jinju frog tracks are also older than any previously reported^8^.

The *Minisauripus* skin impressions, are the first reported from any of the nine known Asian sites. Generally *Minisauripus* tracks from other Korean and Chinese sites are also well preserved, and in some cases associated with other small tetrapod tracks, such as avian theropods^1^ or in some cases in association with raindrop impressions^5^. Arguably, because the *Minisauripus* track assemblage already represents the world’s smallest tridactyl theropod ichnogenus, their presence already implies a minimum standard of preservation in order to be identifiable.

Although there are various scattered reports of skin traces associated with other non-avian theropod and various other dinosaur footprint morphotypes (ichnotaxa), we know of none in which the entire footprint surface registered skin traces throughout a trackway as is the case with CUE JJ_M01-M03. For example, theropod footprints with skin impressions are reported from the Triassic, Jurassic and Cretaceous. The largest such sample was reported^14^ from an assemblage of 19 Late Triassic *Grallator* tracks from Greenland with a mean length of 18.8 cm. These revealed patches with “reticulate” skin impressions on the floor of the tracks and striations on the side walls. The skin traces were referred to as “microtopography” with a reticulate pattern described as “densely packed, loosely hexagonal arrays” that “represent a relatively accurate mold of convex reticulate scales”^14^. Each protuberance in the reticular array could be described as a small polygonal tubercle. These skin protuberances create convexities in the sediment, also referred to as dimples^14^, separated by convex peaks that represented concave troughs between the reticulate scales on the trackmaker’s feet. The average diameter of dimples (=reticulate scales) was given as about 1.0 mm. However, in the Greenland sample skin traces showed a patchy distribution occurring on up to 60–70% of some of the proximal foot pads but on only between 45 and 20% of the distal foot pad traces. However, in no cases were skin traces registered across the floor of the whole footprint^14^.

The Greenland study was one of the more comprehensive dealing with a sample in which the number of skin traces and the proportion of the footprints with skin traces was the highest reported. In contrast, other reports of theropod skin traces from the Jurassic deal with considerably less evidence. For example, a large theropod (*Eubrontes*) track cast from the Early Jurassic, St. George Dinosaur Discovery Site at Johnson Farms, in Utah reveals well preserved skin traces on the proximal pad of digit II^15^. These, traces could also be described as evidence of a reticulate skin texture with densely packed polygons about 1.0–2.0 mm in diameter. However, despite the large sample of well-preserved tracks known from this locality skin traces are comparatively rare and not known to cover entire footprints.

Skin traces have been reported for the heel area of a *Magnoavipes* theropod track cast from the Cretaceous of Colorado^16^. The individual tubercle casts are between 1.0 and 3.0 mm in diameter. These examples demonstrate that reports of theropod tracks with skin impressions are relatively rare, and that the size of polygons in skin ornament increases with track and trackmaker size,

Other tetrapod tracks with skin traces, including those of various non-theropod dinosaurs are too numerous to detail, except to note that virtually all examples are reported as revealing only patchy skin traces^17,18^: i.e., none show coverage of more than a small portion of the footprint. However, there is one unusual Korean occurrence of skin traces pertinent to understanding the preservation of the traces described here. This example involved the claim that the polygonal trace *Paleodictyon*, a well known deep water trace, had been identified in the fluvio-lacustrine Haman Formation, alongside bird tracks^19^. It was later determined that these were sauropod skin impressions associated with extremely shallow footprints that had little or no relief^20,21^. That skin impressions should be clearly registered in such imperceptibly shallow tracks, made by such a large dinosaur, could only be attributed to a substrate in which the uppermost few millimeters was soft and overlying a firm substrate that would not yield to the considerable weight of sauropods. As noted below, similar substrate conditions and interpretations appear to apply to specimens CUE JJ_M01-3.

It is perhaps counter intuitive that large theropod trackmakers would register only patchy skin impressions, whereas the Jinju *Minisauripus* trackmaker registered complete and high resolution skin impression despite its diminutive size. However we know that there are almost 100 known *Minisauripus* tracks and none of those previously reported have skin traces, either in impressions (concave epireliefs) or casts (convex hyporeliefs). This strongly suggests that unusual and optimal preservation conditions must have prevailed in this case.

As noted above, the CUE JJ_M01-3 *Minisauripus* specimens reveal very shallow tracks. Raindrop impressions, which are common in the Jinju Formation are, on the CUE JJ_M01-3 surface flat bottomed, with sharply-defined narrow convex rims less than 1 mm high. This suggest that at the time the light precipitation took place only the uppermost ~1.0 mm of sediment was soft, and that the underlying sandier sediment was firm enough to prevent raindrop impacts from excavating deeper, more concave pits. In all five *Minisauripus* footprints there is only one slender claw trace associated with track TL2. This trace overlaps two raindrop impressions (Fig. 4C), providing evidence that the trackmaker crossed the substrate after these particular raindrops had fallen and registered. At this time substrate conditions were also suitable for the *Cochlichnus* surface trail makers, likely very small (width 0.5 mm) nematodes, to have been active.

The size of the *Minisauripus* trackmaker has been discussed^4–6^, and it is generally agreed that a footprint length (FL) of 2.38 cm converts^22^, into a hip height (*h*) of 10.71 cm (*h* = FL x 4.5 in small theropods). The ratio between hip height (h) and theropod body length has been estimated at ~*h* × 2.63^23^, thus giving an estimated body length of ~28.4 cm. Such a blackbird-sized small animal would have weighed only a few tens of grams. Based on the stride length recorded from the trackway and the values for FL and *h*, we estimate a speed of between 2.27 m/s^22^ and 2.57 m/s^24^ (=8.19 and 9.27 km/h respectively). Under these circumstances, the interaction between a small trackmaker moving at this estimated speed, over the substrate described, we had a rare combination of factors that converged to produce near perfect skin impressions over all footprints in the sample. As in all known *Minisauripus* assemblages the full plantar surface of the foot, toe tip to heel (metatarsal phalangeal pads) was registered. Thus, we must conclude that the trackmaker registered its footprints without sliding and converting the reticulate, tubercular skin ornament pattern into elongated striae such as occur on track walls, and sometimes on track floors. This suggests minimal anterior-posterior, or medial-lateral movement of the foot in contact with the substrate during what has been called touch down, weight-bearing and push off phases, or cycle, of track registrations^23^. Such minimal movement without sliding components implies either that the entire foot registered with a single downward motion, or that if there was a “rolling” heel to toe touchdown motion, creating a history of motion^25^, the foot “stuck” to the substrate without motion between the skin and sediment, throughout the foot registration cycle. Touch down during registration of the foot of such a small animal would have registered very slight impact, perhaps analogous to the leaving of a fingerprint on a damp surface.

After the registration of the *Minisauripus* tracks the stratigraphic evidence shows that another ~1.0 cm of dark fine-grained mud accumulated. During or at the end of this accumulation large pterosaurs left their footprints. These appear to be transmitted underprints that are slightly deeper (~2.0 mm) than the *Minisauripus* tracks, and thus were originally registered on a higher surface. They do not show skin traces, and in at least one case appear to have been the focal point of desiccation cracks, which were presumably propagated after the deposition of the ~1.0 cm mud unit. The slightly greater depth of the pterosaur tracks might be explained by the fact that the trackmakers were larger, heavier animals than the *Minisauripus* trackmaker. The lack of associated pes tracks is a well-known phenomenon in a proportion of known pterosaur track assemblages, and has been explained by the inference that many pterosaurs were front heavy, with less weight carried posteriorly over their pelvic girdles than anteriorly over their more powerful pectoral girdles^26^. It is possible the manus tracks were made by swimming pterosaurs, but this is unlikely as, most pterosaur swim tracks were made by the pes^27,28^

There has been lively debate over the significance of *Minisauripus* and other diminutive tracks. Do they represent small dinosaur species or juveniles of larger species? This debate, touched on only briefly in the 1980s^29^ and 1990s^30^ was stimulated by the new *Minisauripus* discoveries of the last decade^4–6^ and has continued more generally, and inconclusively, to the present time for theropod track assemblages with a wide range of track sizes^31^. The Jinju assemblages are relevant to this debate having recently produced diminutive dromaeosaurid tracks^9^ which appear to correspond to microraptorine species close to the sizes known from the body fossil record. The Jinju *Minisauripus* tracks described here add a ninth important Asia locality to the record bringing the total amended *Minisauripus* track count to ~95, representing an amended estimate of at least 54 trackways^6^. Based on previous syntheses^5,6^ 50 of the 54 trackways (92.6%) fall in the foot length range of 1.1–3.7 cm with only four of these in the FL range 5.0–6.3 cm. [Two larger tracks, (FL 16.1–20.0 cm), listed in the 2012 synthesis^5^ were never proven to have any relationship to *Minisauripus* and so are here removed from the amended data base]. This leaves an estimated 54 trackways of which 44 (=81.5%) are less than 2.9 cm long. Given that no large tracks (>6.1 cm) and few >2.9 cm can presently be assigned to ichnogenus *Minisauripus*, the case for regarding the trackmakers as small species rather than juveniles of large species is the most parsimonious interpretation.

Other important arguments in support of the small species, rather than juvenile trackmaker interpretation, pertain to the major differences in both size and morphology between *Minisauripus* and all other known theropod ichnotaxa reported from the Lower Cretaceous of Korea, and also from China. The entire *Minisauripus* morphometric database is presented in the Supplementary Information, along with the databases for Korean and Chinese theropod ichnogenera *Corpulentapus, Grallator* and *Asianopodus*. Aside from the formal ichnotaxonomic differences enshrined in the literature^1,2,4–6,10^ there is a huge size gap between the largest *Minisauripus* and the smallest representatives of these other three, much larger and morphologically distinct ichnogenera (SI Figs SI1 and SI2).

**Figure 5:**
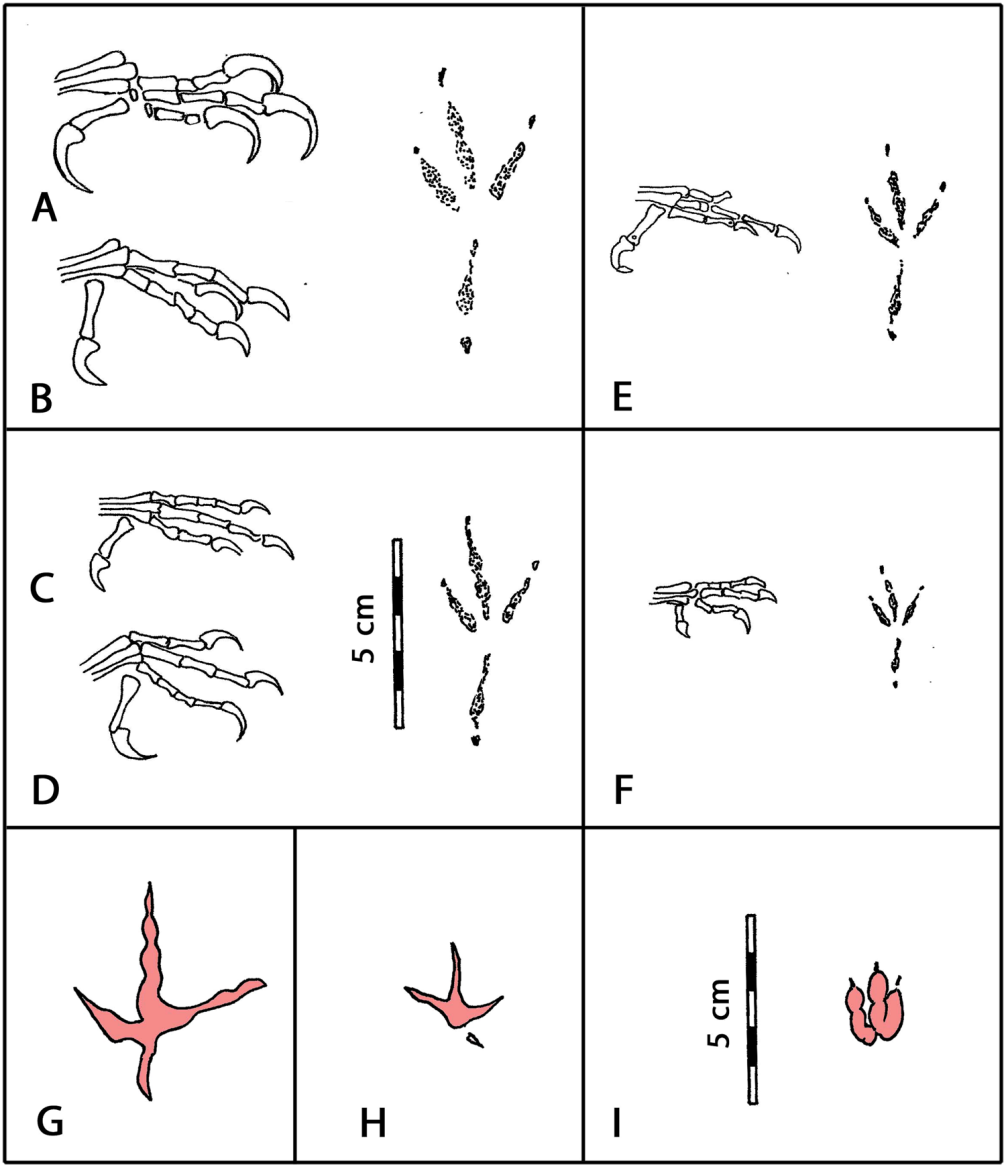
(**A**–**F**) Foot skeletons of Lower Cretaceous birds from China, in order of decreasing size, with reconstructions to match foot skeleton size, based on anisodactyl tracks of modern perching birds with posterior halluxes. (**A**–**D**) *Sapeornis chaoyangensis* (SI ref.^11^.), (**E**) *Shenshiornis primita* (SI ref.^12^.) a subjective synonym of *S*. *chaoyangensis*, according to SI ref.^10^. F: *Eoconfuciusornis zhengi* (SI ref.^9^). Track accompanying A and B based on a modern crow (genus *Corvus*), re-scaled to foot skeleton size in (**C**–**F**). (**G**–**H**) Large and small Cretaceous avian theropod (bird) tracks with halluxes) from Korea: *Jindongornipes* (**G**) and *Koreanaornis* (**H**) with *Minisauripus* (**I**) for comparison. Note that *Minisauripus* is smaller than all other tracks with relatively thick fleshy digits and low digit divarication angles. All images redrawn by MGL, to same scale, in Adobe photoshop (version CS6 www.adobe.com/Photoshop), with modifications to show digits simplified and separated and obstructive material removed for clarity.

Given the small size of *Minisauripus* tracks it is natural to consider whether they might represent birds (small avialans) rather than non-avian theropods. To date, no Lower Cretaceous avialans have been found with feet that would match *Minisauripus*. As discussed in Supplementary Information contemporaneous Lower Cretaceous birds like *Sapeornis* and *Confuciusornis*^32,33^ had well developed, posteriorly-oriented halluxes which would have left diagnostic anisodactyl footprints similar to those of perching birds such as modern passerines (Fig. 5). Based on foot skeletons many of these tracks would have been in the range of 5.0–7.0 cm long, much larger than the vast majority of *Minisauripus* tracks. Moreover, tracks attributed to shorebird like species are well known in the Lower Cretaceous of Korea and China^2^, and are fundamentally different from *Minsauripus*, with slender digit traces, wide digit divarication, inward pes rotation, as well as almost all being considerably larger (Fig. 5: Suppl. Info.).

One interesting similarity however, is that two of the aforementioned Chinese birds show foot integument with reticulate scale patterns^32,33^ similar to those seen in the Jinju *Minisauripus*. However, similarities between avian and non-avian theropod scales in the Mesozoic, while interesting, are no surprise given the close phylogenetic relationships between the two groups (Supplementary Information). Thus they are similar with respect to foot integument scale patterns but markedly different in gross foot morphology.

## Conclusions

The quality of preservation, especially of small tracks of types not previously reported from the Cretaceous^7–9,11^, as well as a large number of excavated specimens still under investigation, mark the Jinju Formation is an example of a Konservat-Lagerstätten defined as a deposit in which body fossil and/or trace fossils show exceptionally good preservation^34,35^.

The *Minisauripus* tracks described here represent the highest resolution of detail yet recorded for any dinosaur skin impressions.

The ninth report of the diminutive theropod track *Minisauripus*, from Jinju City, Korea, is the first to reveal skin impressions. These exquisitely preserved traces of skin with arrays of small reticulate scales (~0.5 mm in diameter) covering the entire plantar surface of the foot, are reminiscent of at least two avialan genera with preserved foot integument. The skin was apparently loose or flexible enough to register in the interdigital areas (hypicies). There have been no other dinosaur or tetrapod footprints reported with well-preserved skin impressions covering the entire track surface in an entire sample. The skin pattern supports attribution of *Minisauripus* to the Theropoda and shows, that the size of polygons in skin ornamentation is proportional to track size.

The tracks, which occur in the Jinju Formation of inferred Aptian age, are the oldest currently known from Korea, and likely as old or older than those reported from China. To date *Minisauripus* is known only from the Lower Cretaceous of East Asia. Moreover, the vast majority of known tracks (~95) are small (~1.0–3.0 cm) suggesting they belong to a small non- avian theropod species and not juveniles, which might be expected to have had larger adult counterparts with the potential to leave larger tracks with diagnostic *Minsauripus* morphology. No such potential “adult” tracks are known despite the increased spatial (regional) and temporal distribution of tracksites. It also appears unlikely, on morphological grounds, or on the basis of known tetrapod ichnofaunas, that *Minsauripus* represents an avian species.

## Supplementary information

Supplementary Info
