# Author Correction: Exquisitely-preserved, high-definition skin traces in diminutive theropod tracks from the Cretaceous of Korea

**DOI:** 10.1038/s41598-019-43832-0

**Published:** 2019-05-30

**Authors:** Kyung Soo Kim, Martin G. Lockley, Jong Deock Lim, Lida Xing

**Affiliations:** 1 Department of Science Education, Chinju National University of Education, 3 Jinnyangho-ro 369beon-gil, Jinju-si, Gyeongnam 52673 Korea; 2 Dinosaur Trackers Research Group, University of Colorado Denver, P.O. Box 173364, Denver, CO 80217 USA; 3 Cultural Heritage Administration, Government Complex-Daejeon, 189, Cheongsa-ro, Seo-gu, Daejon 35208 Korea; 4 School of the earth Sciences and Resources, China University of Geosciences, Beijing, 100083 China

Correction to: *Scientific Reports* 10.1038/s41598-019-38633-4, published online 14 February 2019

This Article contains an error in the Figure legends of Figures 2 and 3 which have been inadvertently reversed.

The correct legend for Figure 2 is:

“(**A**) Map of specimens CUE JJ_M01-3 showing four-track *Minisauripus* trackway, and additional isolated fifth track on small unconnected slab. Map based on counterpart cast of track-bearing surface. Red outline shows part of surface preserved as natural impressions. Pterosaur manus tracks, desiccation cracks (stippled areas), raindrop impressions and invertebrate traces also shown. Compare with Fig. 2 and text for details. (**B**) Shows microstratigraphy of part and counterpart of track-bearing slab. (**C**) Shows four track-trackway with dashed line to highlight steps and pace angulations. See Table 1 for measurements. Map made by M. G. Lockley and K-S Kim with layout created in Canvas X (version, 2017 Build 160, http://www.canvasgfx.com/).”

The correct legend for Figure 3 is:

“(**A**) Counterpart slab CUE JJ_M01 showing trackway with four consecutive *Minisauripus* track casts TL1-TR2. (**B**) Natural impression slab (CUE JJ_M02) showing tracks TL2 and TR2. (**C**) Isolated track specimen (CUE JJ_M03). Compare with Fig. 3. Photographs by K-S Kim and layout created in Canvas X (version, 2017 Build 160, http://www.canvasgfx.com/).”

