# Supplementary material for: Exquisitely-preserved, high-definition skin traces in diminutive theropod tracks from the Cretaceous of Korea
Source: Sci Rep. 2019 Feb 14;9:2039. doi: 10.1038/s41598-019-38633-4 (PMC6375998; doi:10.1038/s41598-019-38633-4)
Supplement: Supplementary file 1 — Supplementary Info [file 41598_2019_38633_MOESM1_ESM.pdf]

Supplementary Information

Exquisitely-preserved, high-definition skin traces in diminutive theropod tracks from the Cretaceous of Korea

Kyung Soo Kim[1], *Martin G. Lockley[2], Jong Deock Lim[3], Lida Xing[4]

[1] *Department of Science Education, Chinju National University of Education, 3 Jinnyangho-ro 369beon-gil, Jinju-si, Gyeongnam, 52673, Korea* kimks@cue.ac.kr

[2] *Dinosaur Trackers Research Group, University of Colorado Denver, P.O. Box 173364, Denver, CO 80217,USA* Martin.Lockley@UCDenver.edu **corresponding author**

[3] *Cultural Heritage Administration, Government Complex-Daejeon, 189, Cheongsa-ro, Seo-gu, Daejon, 35208, Korea* dinostudy@outlook.com

[4] *School of the Earth Sciences and Resources, China University of Geosciences, Beijing 100083, China* Xinglida@gmail.com

N.B. references in superscript [1,4-11, 29- 33] refer to main text. SI references 1-14 , which are not in main text, occur at end of Supplementary Information.

Inferences regarding the *Minisauripus* trackmaker I: juveniles or small species?

As noted in the main text there is ongoing debate over whether small tetrapod tracks, attributed to dinosaurs and other groups represent small species or juveniles of larger species. This intriguing question, first aired in the 1980s [29] and 1990s [30], was revived as a result of the discovery of the presumed non avian theropod track *Minisauripus* at several Chinese and Korean localities [1,4-6,] and has been continued with reference to other non-avian theropod tracks[31]. Although attempting to determine whether diminutive tracks such as *Minisauripus* or *Dromaeosauriformipes*[9] represent the juvenile v. small species is not the main focus of this paper, the continued discovery of small tracks keeps the debate alive.

As discussed in the main text, the juvenile v. small species debate remains inconclusive. However, the weight of evidence, in the form of multiple reports of small tracks, with no reports

of larger morphologically similar tracks, more strongly supports the small species interpretation[6]. The *Minisauripus* track database (Table SI 1- SI 2) representing individual footprints and trackway segments now represents an estimated 54 individuals, of which only four have footprint lengths in excess of 3.7 cms. To test the possibility that *Minisauripus* tracks represent juveniles, rather than a small species we should logically look to the same deposits in which *Minisauripus* occurs for tracks that may represent adults of the same general taxonomic groups: i.e., theropods. We should also consider data from contemporaneous deposits with theropod ichnotaxa that typically co-occur with *Minisauripus*. Although such data exists in the case of most *Minisauripus* tracksites, the co-occurring theropod ichnotaxa are all quite morphologically and ichnotaxonomically distinct, as clearly defined in the literature (SI refs. 1-2)

Table SI 1. Named and unnamed theropod track ichnotaxa associated with *Minisauripus* at known sites in the Lower Cretaceous of China and Korea. Note that there are several Changseon sites in close geographical proximity.

***Minisauripus* site**	**Associated theropod tracks**	**reference**
Emei, China	*Grallator*, *Velociraptorichnus*	main text[1]
Junan, China	*Asianopodus, Dromeaopodus, Velociraptorichnus*	SI ref. 4
Yangmozou China	cf. *Jialingpus*, unnamed theropod	main text[6]
Changseon sites	cf. *Velociraptorichnus, Dromaeosauripus*	main text[5-6]
Sinsu	No associated theropod tracks described or named	main text[5-6]
Gae Je	No associated theropod tracks described or named	main text[5]
Jinju	*Grallator*, *Asianopodus*, *Corpulentapus*	This paper

The first reported *Minisauripus* tracksite from Emei, Sichuan Province, China, yielded only diminutive *Grallator emeiensis* and the didactyl *Velociraptorichnus sichuanensis*[1]. Both tracks are morphologically distinct from *Minisauripus* (SI ref. 2) and cannot possibly be considered representative of adult trackmakers, grown up from individuals that made *Minisauripus* tracks. The same conclusion applies to assemblages from the other Chinese tracksites, Junan in Shandong Province and Yangmozou in Sichuan Province4,6. The Junan site (SI ref. 1) has yielded two didactyl morphotyes (*Dromaeopodus* and *Velociraptorichnus*) that

have absolutely no close phylogenetic or ontogenetic relationship to *Minisauripus*. Likewise the tetradactyl tracks named *Shandongornipes* (SI ref. 3), of presumed avian theropod affinity bears no resemblance *Minisauripus*. The only named tridactyl theropod track from the Junan site is *Asianopodus*, represented by samples from three different horizons, all comprising tracks much large than *Minisauripus*, (FL range 13.0 – 32.0 cm) with diagnostic *Asianopodus* morphology. The FL size gap between the largest *Minisauripus* and smallest *Asianopodus* is 7.0 cm, more than twice the length of the longest known *Minisauripus*. Some smaller *Grallator*-like tracks range in foot length (FL) from 8.0 - 10.0 cm but are morphologically quite distinct from *Minisauripus*. Likewise the smallest non-*Minisauripus* tracks from the Yangmozou site have a mean length of 13.3 cm, with some averaging 20.7 cm. These tracks have also been described as flattened by overburden pressures (SI ref 4), which compromises their utility for comparative analysis.

The most prolific source of *Minisauripus* tracks in Korea has been the Haman Formation at several sites with a small geographical area on Changseon Island[4,5] and nearby Sinsu Island (Table SI 2). With the exception of didactyl tracks[4] almost no theropod tracks have been described in any detail from these localities. The tentative argument that large theropod tracks (FL 16.1 -20.1 cm) "provisionally inferred to represents adults"[5,6] of the *Minisauripus* trackmaker have since been dismissed as weak, on the grounds that there is a gap between (6.1 and 16.1 cm) separating the largest *Minisauripus* and tracks of medium (FL <25 cm) to large (FL> 25 cm) tracks that might remotely "be shown to be morphologically close to *Minisauripus*." The Gae Je locality[5] has revealed no diagnostic larger theropod tracks with which the isolated and poorly preserved *Minisauripus* track could be compared.

In addition to *Minisauripus*, the Jinju Formation has yielded a few *Grallator*, *Asianopodus* and *Corpulentapus*[10] as well as the diminutive dromaeosaur track *Dromaeosauriformipes*[10]. The theropod track assemblage is considered very similar to the theropod-dominated track assemblage from the Huanglonggou site in Shandong Province China, (SI ref 5) particularly in the occurrence of *Corpulentapus* which is currently only known from Huanglonggou and Jinju[10] (Fig. SI 1-2).

The arguments presented above indicate that none of the known *Minisauripus* tracksites have yielded theropod tracks providing persuasive evidence that they might represent adults of the *Minisauripus* trackmaker. On the contrary three of the sites reveal clearly unrelated evidence of didactyl trackmakers.   All of the *Minisauripus* sites that also reveal other tridactyl, theropod tracks have registered tracks that are: a) diagnostically defined as ichnotaxa quite distinct from *Minisauripus*, for example *Grallator* and *Corpulentapus*, b) much larger, at least twice as long as the largest *Minisauripus* tracks, or c) poorly preserved, ichnotaxonomically-ambiguous tracks.

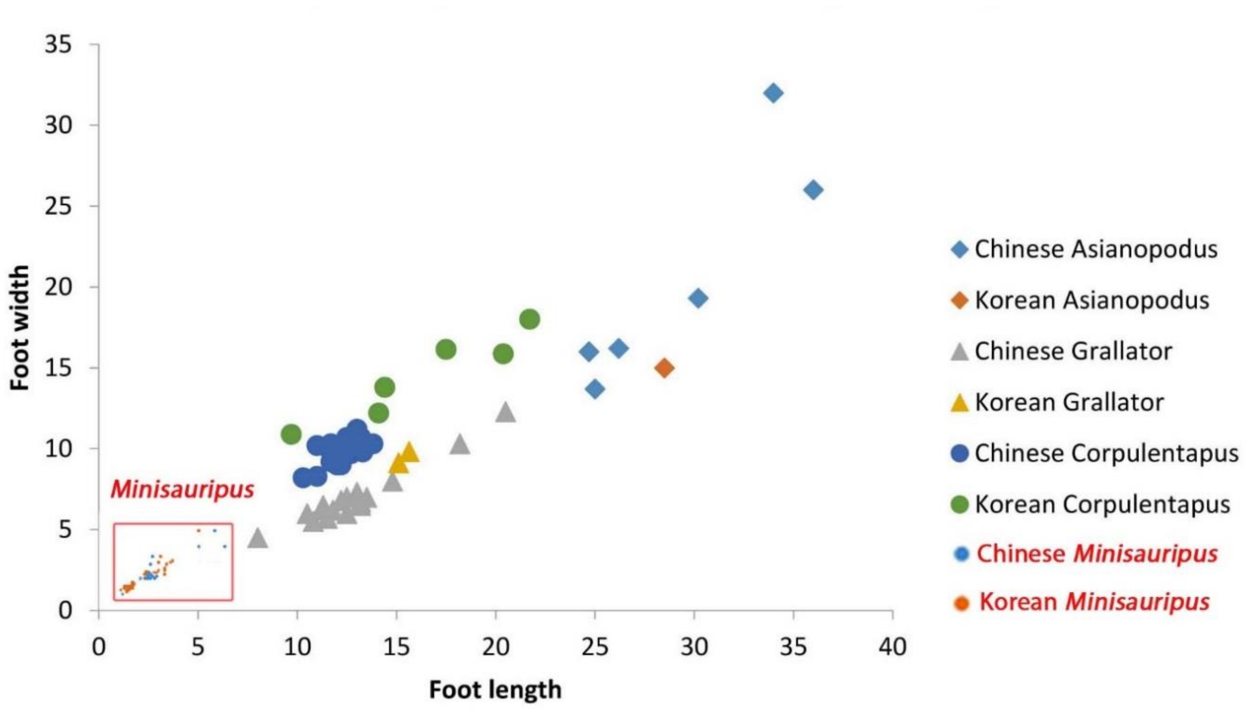

Fig. SI 1. Bivariate plot of *Minisauripus*, *Grallator*, *Asianopodus* and *Corpulentapus* from the Jinju and Haman formations in Korea and contemporaneous deposits in China. Note that there is no size overlap, between *Minisauripus* and the other ichnotaxa. At all known sites the footprint length (FL) size gap between the largest *Minisauripus* and the smallest tridactyl theropod tracks is on the order of three to four times mean *Minisauripus* FL. Compare with Fig. SI 2. Diagrams made by K-S Kand MGL in Adobe photoshop (version CS6 www.adobe.com/Photoshop)   and Canvas X (version, 2017 Build 160, http://www.canvasgfx.com/)

As shown graphically in Figures SI 1 and SI 2, there is no size overlap between *Minisauripus* from either Korea or China and any of the theropod tracks from known *Minisauripus* sites, or from Lower Cretaceous sites from these regions with comparable theropod

track assemblages. As noted above the size differences are in fact of secondary importance in comparison with the established morphological differences that differentiate *Minisauripus* from *Grallator*, *Asianopodus* and *Corpulentapus*[10]. The data used in the compilation of these diagrams includes all the Korean and Chinese data on *Minisauripus* and *Corpulentapus* (Tables SI 1 and SI 2 respectively), and the published data on *Grallator* and *Asianopodus* (SI ref 5). Additional data on Chinese *Asianopodus* and *Grallator* is also available (SI ref 1).

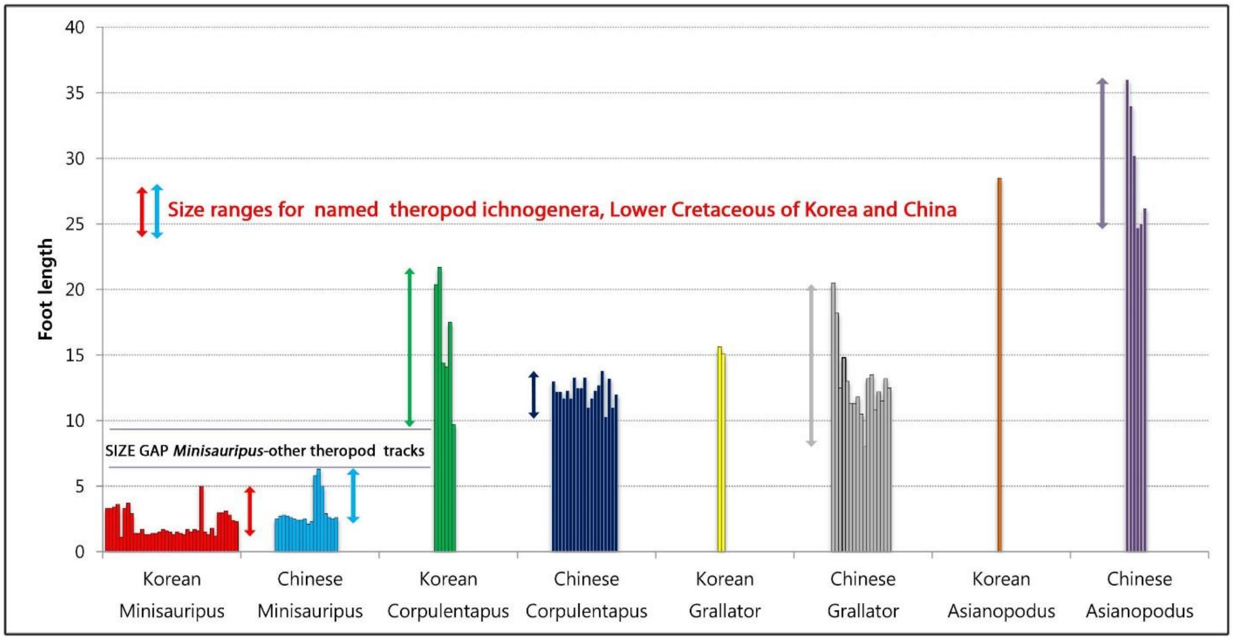

Fig. SI 2. Univariate plot of track length for *Minisauripus*, *Corpulentapus* *Grallator* and *Asianopodus* from Lower Cretaceous formations in Korea and China, showing range of track sizes, and marked size gap between *Minisauripus* and all other theropod ichnotaxa. Compare with Fig. SI 1. Diagrams made by K-S Kand MGL in Adobe photoshop (version CS6 www.adobe.com/Photoshop) and Canvas X (version, 2017 Build 160, http://www.canvasgfx.com/)

No large tracks (FL >6.1 cm) have be assigned to ichnogenus *Minisauripus* with any confidence in any previous studies. The mean size of all *Minisauripus* tracks is ~2.4 cm. Thus, the case articulated previously[6], for regarding the trackmakers as small species rather than juveniles of large species remains the most compelling interpretation.

Table SI 2. *Minisauripus* footprint length (FL), footprint width (FW) and pace length (PL), stride, PL/FL and estimated speed in meters per second (m/s) and kilometers per hour (km/hr)

**Locality** X = reference	**Spec. no**	**FL**	**FW**	**PL**	**stride**	**PL/FL**	**Speed m/s / km/ hr**
Sichuan, China	A 1/2	2.5	1.8	16.2	-	6.48	-
Sichuan, China	A 4 /12/13	2.7	1.7	24.5	-	9.07	-
Sichuan, China	A 6	2.8	1.6	-	-	-	-
Sichuan, China	A 7/9	2.7	2.7	14.0	-	5.18	-
Sichuan, China	A 8/10	2.6	1.6	-	-	-	-
Sichuan, China	A11	2.5	1.6	-	-	-	-
Sichuan, China	A 13	2.4	1.8	-	-	-	-
Sichuan, China	A 14	2.4	1.6	-	-	-	-
Sichuan, China	A 15/16	2.5	1.9	18.4	-	7.36	-
Sichuan, China	A17	2.1	1.6	-	-	-	-
Sichuan, China	A 19	2.3	1.6	-	-	-	-
Shandong, China	A 1/2	5.8	4.0	64.5	-	11.12	-
Shandong, China	B 1/2	6.3	3.2	60.0	-	9.52	-
Shandong, China	C1	5.0	3.2				
Shandong, China	2010 find	2.9	1.7				
Changseon, Korea	KML 1	3.3	2.0				
Changseon, Korea	KML 1	3.3	2.1				
Changseon, Korea	KML 2	3.4	2.3				
Changseon, Korea	KML 2	3.6	2.4	22.5	-	6.25	-
Changseon, Korea	CUE 08 (1001)	1.1	1.0	7.1	-	6.45	1.18 / 4.25
Changseon, Korea	KNUE	3.3	1.8				
Changseon, Korea	KNUE	3.7	2.5				
Changseon, Korea	KNUE	2.9	1.9				
Changseon, Korea	TW1 (1003)	1.4	1.2	8.6	18.4	6.14	-
Changseon, Korea	TW1 (1002)	1.4	1.0	10.8	-	7.71	3.73 /10.36

Changseon, Korea	TW2	1.7	1.1	21.0	42.1	12.35	-
Changseon, Korea	TW3	1.3	1.2	15.7	-	12.07	-
Changseon, Korea	TW4	1.3	1.1	23.4	-	18.0**	-
Changseon, Korea	TW5	1.4	1.0	18.0	-	12.85	-
Changseon, Korea	TW6	1.4	1.0	17.8	-	12.71	-
Changseon, Korea	T1	1.5	1.1	-	-	-	-
Changseon, Korea	T2	1.7	1.3	-	-	-	-
Changseon, Korea	T3	1.6	1.1	-	-	-	-
Changseon, Korea	T4	1.5	1.1	-	-	-	-
Changseon, Korea	T5	1.3	1.1	-	-	-	-
Changseon, Korea	T6	1.5	1.1	-	-	-	-
Changseon, Korea	T7	1.4	0.9	-	-	-	-
Changseon, Korea	T8	1.3	1.1	-	-	-	-
Changseon, Korea	T9	1.7	1.2	-	-	-	-
Changseon, Korea	T10	1.5	1.2	-	-	-	-
Changseon, Korea	T11	1.7	1.4	-	-	-	-
Sinsu, Korea	-	1.6	1.2	6.0	11.7	3.75*	0.47 / 1.69
Gae Je	-	5.0	4.0				
Changseon, Korea	T1 (1002)	1.5	1.0	-	-	-	-
Changseon, Korea	T2 (1002)	1.3	-	-	-	-	-
Changseon, Korea	T3 (1002)	1.8	1.3	-	-	-	-
Changseon, Korea	T4 (1002)	1.2	0.8	-	-	-	-
Yangmozou, China	YMZ-T1	2.6	1.8	20.1	40.4	7.73	2.12 /7.73
Yangmozou, China	YMZ-T2	2.5	1.7	37.5	75.0	15.00	6.24 /22.46
Yangmozou, China	YMZ-T3	2.6	2.3	27.0	53.8	10.34	3.42 /12.31
Sangcheon Korea	CUE SC-002	3.0	2.4				
Sangcheon Korea	UCM 214.313	3.0	2.0				
Sangcheon Korea	UCM 214.314	3.1	2.7				
Sangcheon Korea	UCM 214.315	2.8	1.9				
Jinju Korea	M01-M02	2.38	1.93	20.03	39.6	8.41	2.57 /9.27
Jinju Korea	M03	2.3	1.8				

Table SI 3. *Corpulentapus* tracks from Lower Cretaceous, Yangjiazhang Formation, Huanglonggou, China (C1-C19)[X] and Jinju Formation, Korea (red). JPFM = Jinju Pterosaur Footprint Museum (this paper, TW2 and TTD (after ref X).

Trackway #	Footprint Length (N)	Footprint Width (N)	Pace length (N)	Stride (N)
C1	13.0 (3)	11.2 (3)	62.7 (3)	123.5 (2)
C2	12.2 (3)	9.0 (3)	60.2 (3)	121.0 (2)
C3	12.2 (3)	10.0 (3)	58.0 (3)	115.0 (2)
C4	11.7 (3)	10.3 (3)	70.3 (2)	140.0 (1)
C5	12.3 (3)	10.3 (3)	62.3 (2)	126.0 (2)
C6	11.7 (3)	9.2 (3)	59.8 (2)	120.0 (1)
C7	13.3 (3)	9.8 (3)	74.8 (3)	148.5 (2)
C8	12.5 (3)	10.3 (3)	66.0 (2)	132 (1)
C9	12.5 (3)	10.7 (3)	68.5 (2)	137 (1)
C10	13.3 (2)	10.5 (2)	74.0 (1)	_
C11	11.0 (2)	8.3 (2)	71.0 (1)	-
C12	11.7 (3)	9.8 (3)	47.0 (2)	94.0 (1)
C13	12.3 (3)	10.3 (3)	58.2 (3)	117.0 (2)
C14	12.7 (3)	9.7 (3)	60.5 (2)	121.0 (1)
C15	13.8 (2)	10.3 (2)	71.0 (1)	-
C16	10.3 (3)	8.2 (3)	51.5 (2)	103.0 (1)
C17	13.2 (3)	10.7 (3)	72.5 (2)	145.0 (1)
C18	11.0 (3)	10.2 (3)	58.5 (2)	117 (1)
C19	12.0 (1)	9.0 (1)	57.0 (1)	-
**Chinese Means**	**12.24 (19)**	**9.92 (19)**	**63.36 (19)**	**124.00 (15)**
TW 2	20.38 (5)	15.88 (5)	95.1	189.9
HTD	21.7	18.0	-	
JPFM 1	14.4 (2)	13.8	-	
JPFM 2	14.1 (6)	12.2	63.9 (4)	
JPFM 3	17.5	16.15	-	
JPFM 4	9.7 (4)	10.9 (4)	43.0 (3)	
**Korean means**	**16.3 (6)**	**12.8 (6)**	**70.0 (3)**	

Inferences regarding the *Minisauripus* trackmaker II: avian or non-avian theropod.

Minisauripus has been attributed to a non-avian theropod, due to the relatively wide or 'fleshy' digit traces, with very low digit divarication angles. These contrast with almost all known Cretaceous avian theropod (bird) tracks which show wide digit divarication angles and slender toe traces, reminiscent of shorebirds (main text Fig. 5, SI refs 6-7). Many bird tracks also reveal hallux traces, and inward rotation of the pes, features not seen in *Minisauripus*.

Contemporaneous Lower Cretaceous birds like *Sapeornis chaoyangensis* [32] (and purported synonyms *Didactylornis* and *Shenshiornis*) together with *Jeholornis* and *Confuciusornis*, [33] (SI refs 8-14) had well developed, posteriorly-oriented halluxes (or hallicies) which indicate a perching adaptation and a typical anisodactyl foot which probably would have generated diagnostic anisodactyl footprints if the birds registered tracks on suitable substrates (Fig. SI 3). However, the vast majority of fossil avian theropod (bird) tracks represent shorebird-like footprints with wide digit divarication and shorter hallux traces than those registered by perching birds such as passerines (SI refs. SI 6-7).

Some saperornid bird fossils display feet with well preserved integument (skin) traces [32]. A particularly striking example is provided by a juvenile specimen of *S. chaoyangensis* from the Lower Cretaceous Jiufotang Formation, of Liaoning Province, China, which reveals and integument of black carbonized matter around the whole body including all parts of the feet except the distal claws[32]. Close inspection of this integument indicates that it only reveals regular scaly texture around the foot pads, on a foot that would have been ~ 6.0 cm long with hallux extended. The texture is described as "round, pentagonal or suboval scales" with an "average diameter of about 0.36 mm[32] (p. 35): compare with ~0.5 mm in *Minisauripus*. These scales are inferred to have "facilitated gripping branches" when perching. Reticulate scales (diameter ~ 0.35- 0.5 mm) have also been described in the foot of *Confuciusornis*,[33] and interpreted as being more flexible than overlapping scutellate scales.

It is not surprising that avian and non-avian theropods would have similar skin texture patterns, since both belong to closely related clades. However, as illustrated here (main text Figs. 4,5), the similarity in texture occurs in association with feet and footprints with quite different morphologies. Thus, reticulate skin texture is developed independent of gross foot morphology, in avian and non-avian theropods, and we cannot be attribute *Minisauripus* to a bird.

Supplementary Information References

1. Li, R., Lockley, M. G., Matsukawa, M. & Liu, M. Important Dinosaur-dominated footprint assemblages from the Lower Cretaceous Tianjialou Formation at the Houzuoshan Dinosaur Park, Junan County, Shandong Province, China. *Cretaceous Research.* **52**, 83-100 (2015).

2. Lockley, M. G. New perspectives on morphological variation in tridactyl footprints: clues to widespread convergence in developmental dynamics. *Geological Quarterly*. **53**, 415-432 (2009).

3. Lockley, M. G., Li, R., Harris, J., Matsukawa, M. & Mingwei, L. Earliest zygodactyl bird feet: evidence from Early Cretaceous Road Runner-like traces. *Naturwissenschaft*. **94**, 657-665 (2007).

4. Lockley, M. G. & Xing, L. Flattened fossil footprints: implications for paleobiology. *Palaeogeography, Palaeoclimatology, Palaeoecology*. **426,** 85-94 (2015).

5. Lockley, M.G., Li, R., Matsukawa, M., Xing, L. Li, J., Liu, M., and Xing, X. Tracking the yellow dragons Implications of China's largest dinosaur tracksite (Cretaceous of the Zhucheng area, Shandong Province, China). *Palaeogeography, Palaeoclimatology, Palaeoecology*, **423**, 62-79 (2015).

6. Lockley, M. G., Yang, S-Y., Matsukawa, M., Fleming, F. and Lim, S-K. The Track Record of Mesozoic Birds: Evidence and Implications. *Philosophical Transactions Royal Society London*, **336**, 113-134 (1992).

7. Lockley, M. G., Lim, J. D., Kim, J-Y. Kim K-S. Huh, M. Hwang, K.G,. Tracking Korea’s early birds: ichnological insights into avian evolution and behavior. *Ichnos* **19**, 17-27 (2012).

8. Zhou, Z. and Zhang, F. A long tailed, seed eating bird from the Early Cretaceous of China. *Nature*, **418,** 405-409 (2002).

9. Zhou, Z. and Zhang, F. Mesozoic birds of China – a synoptic review. Vertaberata PalAsiatica, **44**, 74-98. (2006)

10. Gao, C., Chiappe. L. M., Zhang, F., Pomeroy, D. L., Shen, C., Chinsamy, A., & Walsh. M. O. A subadult specimen of the Early Cretaceous bird *Sapeornis chaoyangensis* and a taxonomic reassessment of sapeornithids, Journal of Vertebrate Paleontology, 32:5, 1103-1112, DOI: 0.1080/02724634.2012.693865 (2012)

11. Zhang, F., Zhu, Z., and Benton, M. J. A primitive confuciusornithid bird from China and it implications for early avain flight. *Science in China D: Earth Science* **51**, 625-639 (2008).

12. O'Connor, J. K., Sun, C. , Xu, X., Wang, X., & and Zhou, Z. A new species of *Jeholornis* with complete caudal integument, *Historical Biology*, **24**, 29-41, (2012) DOI: 10.1080/08912963.2011.552720

13. Yuan, C. A new Genus and species of Sapeornthidae from the Lower Cretaceous of Western Liaoning, China. *Acta Geological Sinica*, **82**, 48-55 (2008).

14. Hu, D., Li, L. Hou. L., and Xu. X. A new sapeornid bird from China and its implications for Early Avian evolution, *Acta Geological Sinica* **84**, 472-482 (2010)
